# Moral injury and the four pillars of bioethics

**DOI:** 10.12688/f1000research.19754.1

**Published:** 2019-07-26

**Authors:** Thomas F Heston, Joshuel A Pahang

**Affiliations:** 1 Medical Education and Clinical Sciences, Elson S. Floyd College of Medicine, Washington State University, Spokane, WA, 99210-1495, USA

**Keywords:** moral injury, burnout, bioethics

## Abstract

Health care providers experience moral injury when their internal ethics are violated. The routine and direct exposure to ethical violations makes clinicians particularly vulnerable to harm. The fundamental ethics in health care typically fall into the four broad categories of patient autonomy, beneficence, nonmaleficence, and social justice. Patients have a moral right to determine their own goals of medical care, that is, they have autonomy. When this principle is violated, moral injury occurs. Beneficence is the desire to help people, so when the delivery of proper medical care is obstructed for any reason, moral injury is the result. Nonmaleficence, meaning do no harm, has been a primary principle of medical ethics throughout recorded history. Yet today, even the most advanced and safest medical treatments all are associated with unavoidable, harmful side-effects. When an inevitable side-effect occurs, not only is the patient harmed, the clinician also suffers a moral injury. Social injustice results when patients experience suboptimal treatment due to their race, gender, religion, or other demographic variables. While moral injury occurs routinely in medical care and cannot be entirely eliminated, clinicians can decrease the prevalence of injury by advocating for the ethical treatment of patients, not only at the bedside, but also by addressing the ethics of political influence, governmental mandates, and administrative burdens on the delivery of optimal medical care. Although clinicians can strengthen their resistance to moral injury by deepening their own spiritual foundation, that is not enough. Improvements in the ethics of the healthcare system as a whole are necessary in order to improve medical care and decrease moral injury.

## Introduction

Moral injury occurs when a person experiences an immoral event that disrupts their fundamental moral integrity. Injuries can be self-inflicted by intentionally doing something wrong or come about as collateral damage through observation of a real or perceived action that violates an internal sense of right and wrong. Those suffering from moral injury have a disruption of their sense of morality, with consequences impacting their capacity to behave in a moral manner. The injury reduces their capacity to think of themselves as a moral, good person ( Yan, 2016).

The term moral injury was introduced initially to describe the reaction of military veterans to the participation in or observation of profound ethical transgressions occurring during wartime ( Shay & Munroe, 1999). The diagnosis of moral injury in veterans relies on the presence of three factors: a betrayal of what is right, which is carried out by someone who holds legitimate authority (e.g. a leader), and occurs in a high stakes situation ( Shay, 2014). The diagnosis of moral injury, however, has not been limited to those exposed to the atrocities of war. It has also been evaluated in refugees, health care workers, and adolescents transitioning to adults ( Chaplo *et al.*, 2019). In these diverse groups, while moral injury is recognized as a distinct entity from other psychological conditions, such as post-traumatic stress disorder, the diagnosis relies on poorly defined, generalized criteria, which is very similar to that used for combat veterans. While symptom scales have been developed for military personnel, adolescents, and refugees, no specific diagnostic criteria exist for health care workers ( Chaplo *et al.*, 2019; Koenig *et al.*, 2018; Nickerson *et al.*, 2018).

The optimal treatment of moral injury, just like the diagnosis of moral injury, remains unclear. Proposals to treat moral injury in medical professionals include participation in support groups, building up personal character, and personal reflection by keeping a diary. The inclusion of standard treatments for post-traumatic stress disorder in veterans suffering from moral injury has also been proposed.

A maxim of medicine is that a correct diagnosis is half the cure. In the case of moral injury as it specifically applies to medical professionals, we propose that a violation of the four pillars of bioethics forms the foundation of the diagnosis. We propose a framework for moral injury in health care based upon the four pillars of bioethics ( Beauchamp, 2006). These pillars are patient autonomy, beneficence, nonmaleficence, and social justice. They serve as an effective foundation for evaluating moral behavior in medicine. Our framework clarifies the meaning of moral injury in medicine. When a physician, nurse, or other health care provider participates in, or witnesses a violation of, one or more of these core principles, moral injury occurs. Treatment strategies focused on repairing the breach of these principles of morality in health care may be the best way to heal the injury. Improving the recognition of and reflection upon the moral stressors that clinicians encounter in their practice may prevent moral injury from progressing further. This framework will help more clearly define moral injury in medical professionals, allowing the development of treatment specific to those working in health care.

## Patient autonomy

The principle of respect for autonomy holds that each person with capacity has the right to make their own decisions, and providers have a moral obligation to respect this right. In the clinician-patient relationship, patient autonomy can be especially vulnerable. This principle is often at the forefront of ethical concerns in health care ( Entwistle *et al.*, 2010); ( Stammers, 2015).

Compromising patient autonomy can result in moral injury, regardless of whether or not the perceived event is a true violation. For example, children presenting to the emergency department may openly voice a desire to not get an injection or an intravenous line. Although it is recognized that the decision of the legal caregiver overrides that of a young child, the perception of compromised autonomy can result in moral injury. Although the reason for the injection or intravenous line is medically indicated, the action nevertheless is against the will of the child. Logically, we know children will cry and object to many medical treatments. Still, whenever possible, it is recommended to obtain consent from both the child and the parent. Consent to treatment requires permission from the legal representative of the child, and if possible, assent from the child as well ( Tait & Hutchinson, 2018). The accumulation of such experiences that challenge the clinician’s duty to respect patient autonomy may eventually lead to moral injury.

## Nonmaleficence

The principle of nonmaleficence is captured by the Latin maxim, *primum non nocere:* “above all, do no harm.” It has been estimated that medical error is the third leading cause of death in the United States ( Makary & Daniel, 2016). While the potential to reduce these errors is debated, common preventable harms include medication adverse events, central line infections, and thromboembolisms ( Nabhan *et al.*, 2012). With increasing ability to treat patients comes increasing opportunity to harm patients as systems become more complex. Most clinicians are very aware and regularly reminded of these statistics, however, the seemingly futile efforts to try and reduce the incidence of these harms is troublesome and can contribute to moral injury. Bureaucratic and administrative interference, well intended or not, can hamper efforts by physicians and nurses to decrease harm, leading to moral injury and a sense of powerlessness.

## Beneficence

With the many opportunities to harm a patient in mind, we must also remember that patients come to clinicians in search of improvement or restoration of their health, which leads to the principle of beneficence. The commitment to helping others is the driving force amongst health care workers and to accomplish this goal there must be a net benefit over harm ( Gillon, 1994). Decisions on diagnostic pathways, treatment plans and societal policies all must balance the benefit versus harms, and these balances also must be made in context of the patient’s values.

Beneficence, when compromised, creates numerous conflicts in medicine that can result in moral injury. When the cost of proper medical care exceeds the ability of an individual patient to pay, beneficence can be compromised. Pharmaceutical pricing is a common cause of this moral compromise. For example, many patients with atrial fibrillation will benefit from changing their warfarin prescription to a newer, direct oral anticoagulant such as apixaban. However, the up-front price of the newer medication prohibits them from changing, even though the total financial cost of the newer medication is estimated to be lower due to fewer medical complications ( Gupta *et al.*, 2018). Beyond the financial impact, the negative impact upon the patient’s health can be devastating. Compromising the principle of beneficence occurs when the patient is unable to take the best medication because of financial limitations. Although the medical complications from the older medication will ultimately cost more money, the hard reality is that patients will take the cheaper medication because they cannot afford the up-front costs of the newer, better medication.

## Social justice

The final pillar of bioethics is social justice. Justice demands that limited resources be distributed fairly, and that patients not be discriminated against due to any number of demographic variables such as race, religion, gender identity, sexual orientation, age, or cultural background. Moral injury occurs when these ideals conflict with the hard reality of medical care where discrimination does occur, primarily along socioeconomic lines.

These complex socioeconomic disparities cause moral injury because clinicians know what their patients need and find the economic barriers to needed care to be illogical, unnecessary, and capricious. They know that not getting that nursing home bed placement will result in a bad outcome, often at a much higher cost. They know that not getting a patient with a substance use disorder necessary treatment will ultimately cost more to society, although the health care plan may save money. They have seen first-hand the elderly family member decide they would rather die than leave a large medical bill for their surviving relatives. Witnessing these events on a regular basis doesn’t cause burnout, it causes moral injury.

Medical professionals working in medical systems and countries that rely on privately funded insurance may also experience a constant violation of the principle of social justice. For example, one study comparing a population with universal medical insurance found disparities in the care given to racial and ethnic minorities to be greatly decreased or even eliminated ( Chaudhary *et al.*, 2018). A similar study found that universal medical insurance ameliorated socioeconomic disparities in mortality ( Veugelers & Yip, 2003). Medical professionals working in private medical insurance systems who know about and trust such research studies may experience a persistent low-grade violation of their bioethics. This, over time, may progress to symptomatic moral injury. The primary means of addressing such issues would be meaningful involvement in improving the larger health care system.

## Conclusion

Moral injury occurs when there is a disruption in an individual’s sense of personal morality and capacity to behave in a just manner. It is a common occurrence in medicine because of ongoing violations of bioethics that have become an intrinsic part of the healthcare system. The prevention of moral injury is accomplished by decreasing violations of the four pillars of bioethics whenever possible. Patients deserve autonomy, and we can give this to them. Although we cannot always help our patients as much as we would like, we can always help them in at least some way. We can be vigilant when taking measures to increase patient safety and decrease harm. With a firm understanding of the basic principles of bioethics, medical professionals can become more adept at identifying and reflecting upon moral violations in the workplace. This recognition helps prevent recurrent moral injury, decreases burnout, and can help to heal previous injuries.

## Data availability

No data are associated with this article.

## References

[ref-1] Beauchamp TL : The ‘four principles’ approach to health care ethics. In: Ashcroft, R.E., Dawson, A., Draper, H., and McMillan, J. R. eds. *Principles of health care ethics*. Chichester, UK: John Wiley & Sons, Ltd.2006;3–10. 10.1002/9780470510544.ch1

[ref-2] Chaplo SD Kerig PK Wainryb C : Development and validation of the moral injury scales for youth. *J Trauma Stress.* 2019;32(3):448–458. 10.1002/jts.22408 31162746

[ref-3] Chaudhary MA Sharma M Scully RE : Universal insurance and an equal access healthcare system eliminate disparities for Black patients after traumatic injury. *Surgery.* 2018;163(4):651–656. 10.1016/j.surg.2017.09.045 29221878

[ref-4] Entwistle VA Carter SM Cribb A : Supporting patient autonomy: the importance of clinician-patient relationships. *J Gen Intern Med.* 2010;25(7):741–745. 10.1007/s11606-010-1292-2 20213206 PMC2881979

[ref-5] Gillon R : Medical ethics: four principles plus attention to scope. *BMJ.* 1994;309(6948):184–188. 10.1136/bmj.309.6948.184 8044100 PMC2540719

[ref-6] Gupta K Trocio J Keshishian A : Real-World Comparative Effectiveness, Safety, and Health Care Costs of Oral Anticoagulants in Nonvalvular Atrial Fibrillation Patients in the U.S. Department of Defense Population. *J Manag Care Spec Pharm.* 2018;24(11):1116–1127. 10.18553/jmcp.2018.17488 30212268 PMC10398049

[ref-7] Koenig HG Ames D Youssef NA : The Moral Injury Symptom Scale-Military Version. *J Relig Health.* 2018;57(1):249–265. 10.1007/s10943-017-0531-9 29196962

[ref-8] Makary MA Daniel M : Medical error-the third leading cause of death in the US. *BMJ.* 2016;353:i2139. 10.1136/bmj.i2139 27143499

[ref-9] Nabhan M Elraiyah T Brown DR : What is preventable harm in healthcare? A systematic review of definitions. *BMC Health Serv Res.* 2012;12:128. 10.1186/1472-6963-12-128 22630817 PMC3405467

[ref-10] Nickerson A Hoffman J Schick M : A Longitudinal Investigation of Moral Injury Appraisals Amongst Treatment-Seeking Refugees. *Front Psychiatry.* 2018;9:667. 10.3389/fpsyt.2018.00667 30618859 PMC6305427

[ref-11] Shay J : Moral injury. *Psychoanalytic Psychology.* 2014;31(2):182–191. 10.1037/a0036090

[ref-12] Shay J Munroe J : Group and milieu therapy for veterans with complex posttraumatic stress disorder.In: Saigh, P. A. and Bremner, J. D. eds. *Posttraumatic stress disorder: A comprehensive text.*Boston: Allyn and Bacon,1999;391–413. Reference Source

[ref-13] Stammers T : The evolution of autonomy. *New Bioeth.* 2015;21(2):155–163. 10.1179/2050287715Z.00000000070 27124963

[ref-14] Tait AR Hutchinson RJ : Informed Consent Training in Pediatrics-Are We Doing Enough? *JAMA Pediatr.* 2018;172(3):211–212. 10.1001/jamapediatrics.2017.4088 29356830

[ref-15] Veugelers PJ Yip AM : Socioeconomic disparities in health care use: Does universal coverage reduce inequalities in health? *J Epidemiol Community Health.* 2003;57(6):424–428. 10.1136/jech.57.6.424 12775787 PMC1732477

[ref-16] Yan GW : The invisible wound: moral injury and its impact on the health of operation enduring freedom/operation iraqi freedom veterans. *Mil Med.* 2016;181(5):451–458. 10.7205/MILMED-D-15-00103 27136652

