# Moral injury and the four pillars of bioethics

**DOI:** 10.12688/f1000research.19754.3

**Published:** 2023-10-09

**Authors:** Thomas F Heston, Joshuel A Pahang

**Affiliations:** 1 Medical Education and Clinical Sciences, Elson S. Floyd College of Medicine, Washington State University, Spokane, WA, 99210-1495, USA

**Keywords:** moral injury, burnout, bioethics

## Abstract

Health care providers experience moral injury when their internal ethics are violated. The routine and direct exposure to ethical violations makes clinicians vulnerable to harm. The fundamental ethics in health care typically fall into the four broad categories of patient autonomy, beneficence, nonmaleficence, and social justice. Patients have a moral right to determine their own goals of medical care, that is, they have autonomy. When this principle is violated, moral injury occurs. Beneficence is the desire to help people, so when the delivery of proper medical care is obstructed for any reason, moral injury is the result. Nonmaleficence, meaning do no harm, has been a primary principle of medical ethics throughout recorded history. Yet today, even the most advanced and safest medical treatments are associated with unavoidable, harmful side effects. When an inevitable side effect occurs, the patient is harmed, and the clinician is also at risk of moral injury. Social injustice results when patients experience suboptimal treatment due to their race, gender, religion, or other demographic variables. While minor ethical dilemmas and violations routinely occur in medical care and cannot be eliminated, clinicians can decrease the prevalence of a significant moral injury by advocating for the ethical treatment of patients, not only at the bedside but also by addressing the ethics of political influence, governmental mandates, and administrative burdens on the delivery of optimal medical care. Although clinicians can strengthen their resistance to moral injury by deepening their own spiritual foundation, that is not enough. Improvements in the ethics of the healthcare system as a whole are necessary to improve medical care and decrease moral injury.

## Introduction

Moral injury occurs when a person experiences an immoral event that disrupts their fundamental moral integrity. Injuries can be self-inflicted by intentionally doing something wrong or coming about as collateral damage through observation of an actual or perceived action that violates an internal sense of right and wrong. Those suffering from moral injury have a disruption of their sense of morality, with consequences impacting their capacity to behave morally. The injury reduces their capacity to think of themselves as a moral, good person ( Yan, 2016).

Unlike post-traumatic stress disorder (PTSD), which is typically associated with the experience of physical harm or threat, moral injury involves the reaction of military veterans to the participation in or observation of profound ethical transgressions occurring during wartime ( Shay & Munroe, 1999). It is the lasting psychological, biological, spiritual, behavioral, and social effects of perpetrating, failing to prevent, or bearing witness to troubling acts that violate deeply held moral beliefs and expectations ( Litz *et al*., 2009).

The diagnosis of moral injury in veterans relies on three factors: a betrayal of what is right, carried out by someone who holds legitimate authority (e.g., a leader) and occurs in a high-stakes situation ( Shay, 2014). Furthermore, it is not just an incidental injury but an ongoing syndrome resulting from the physical, psychological, social, and spiritual harm from such moral transgressions ( Jinkerson, 2016).

Moral injury, however, has not been limited to those exposed to the atrocities of war. It has also been evaluated in refugees, healthcare workers, and adolescents transitioning to adults ( Chaplo *et al*., 2019). In these diverse groups, while moral injury is recognized as a distinct entity from other psychological conditions, such as post-traumatic stress disorder, the diagnosis relies on poorly defined, generalized criteria similar to that used for combat veterans. While minor ethical dilemmas and lapses in judgment are commonplace, these alone may not be sufficient to cause moral injury. The moral violations precipitating moral injury tend to be severe transgressions that fundamentally undermine one's moral integrity. Symptom scales have been developed for military personnel, adolescents, and refugees, but no specific diagnostic criteria exist for healthcare workers ( Chaplo *et al*., 2019; Koenig *et al*., 2018; Nickerson *et al*., 2018).

The optimal treatment of moral injury remains unclear. Proposed approaches to treating moral injury in medical professionals include participating in support groups, building personal character through reflection, keeping a diary, and incorporating PTSD treatments used for veterans. However, the effectiveness of these approaches is unknown. A recent scoping review ( Jones *et al*., 2022) highlights that while interest in moral injury has grown exponentially in recent years, high-quality empirical studies on the effectiveness of interventions are still lacking. Compassion-focused therapy, eye movement desensitization and reprocessing, schema therapy, and mindfulness have all been utilized to treat moral injury. However, there is currently no consensus on the optimal treatment approach, with providers frequently blending multiple therapies. The review also noted that providers needed 12–16 sessions to adequately treat the moral injury, longer than some standardized PTSD protocols, reinforcing that moral injury may require different treatment considerations than PTSD. Finally, there is a need for greater clinician education on the diagnosis of moral injury as well as outcome measures to properly evaluate the spectrum of psychosocial and spiritual impacts of interventions. Developing and empirically testing interventions targeted specifically to moral injury in healthcare workers remains an important research priority.

A maxim of medicine is that a correct diagnosis is half the cure. In the case of moral injury, as it specifically applies to medical professionals, we propose that violating the four pillars of bioethics forms the foundation of the diagnosis. We propose a framework for moral injury in health care based on the four pillars of bioethics ( Beauchamp & Childress, 2019). These pillars are patient autonomy, beneficence, nonmaleficence, and social justice. They serve as an effective foundation for evaluating moral behavior in medicine. Our framework clarifies the meaning of moral injury in medicine. Moral injury occurs when a physician, nurse, or other health care provider participates in or witnesses a significant violation of one or more of these core principles. Treatment strategies focused on repairing the breach of these principles of morality in health care may be the best way to heal the injury. Improving the recognition of and reflection upon the moral stressors clinicians encounter in their practice may prevent moral injury from progressing. This framework will help more clearly define moral injury in medical professionals, allowing the development of treatment specific to those working in health care.

Moral residue, failure, and distress are all related to moral experiences, but they have distinct meanings. Moral residue refers to the lingering emotional and psychological impact of being involved in or witnessing morally challenging situations. It is the residue left behind after a moral dilemma or ethical conflict. In contrast, moral failure refers to intentionally or unintentionally violating one's moral principles or ethical standards. It is a personal failure to uphold the values one believes in ( Tessman, 2020). Moral distress, meanwhile, is the psychological and emotional anguish that arises from being unable to act following one's moral beliefs due to external constraints or conflicting obligations. It is the self-directed distress experienced in response to perceived involvement in a situation that is morally undesirable ( Campbell *et al*., 2016). These concepts are related to, but distinct from, moral injury, which refers to profound and lasting psychological and spiritual harm resulting from acts of moral transgression, betrayal, or witnessing atrocities ( Boudreau, 2011). Moral injury involves profound disruption across biological, psychological, social, and spiritual dimensions. As outlined by Hodgson and Carey ( Hodgson & Carey, 2017), moral injury should be conceptualized as an "eclectic of injuries" spanning physiological, emotional, relational, and religious/spiritual dimensions. Each dimension has associated symptoms, some overlapping and some unique. Fully capturing the complex impacts requires acknowledging moral injury as a bio-psycho-social-spiritual syndrome.

While minor ethical dilemmas and violations may commonly occur in healthcare, the type of profound, grievous moral transgression required to cause moral injury is less frequent. Moral injury results explicitly when there is a severe betrayal of moral beliefs and ethical standards, not just an everyday lapse or poor judgment. The moral violations that precipitate moral injury are severe enough to fundamentally challenge one’s moral integrity and capacity for moral behavior. Examples include participating in dishonest billing practices, knowingly covering up a medical error, or conducting unwanted procedures. Moral injury aligns with egregious breaches, not minor inconsistencies in morality. This article focuses on these severe transgressions that entirely violate the fundamental bioethical pillars and give rise to the syndrome of moral injury.

## Patient autonomy

The principle of respect for autonomy holds that each person with capacity has the right to make their own decisions, and providers have a moral obligation to respect this right. In the clinician-patient relationship, patient autonomy can be especially vulnerable. This principle is often at the forefront of ethical concerns in health care ( Entwistle *et al*., 2010; Stammers, 2015).

A significant compromise in patient autonomy can result in moral injury, regardless of whether or not the perceived event is an actual violation. For example, children presenting to the emergency department may openly voice a desire not to get an injection or an intravenous line. Although it is recognized that the decision of the legal caregiver overrides that of a young child, the perception of compromised autonomy raises concern for moral injury. Although the reason for the injection or intravenous line is medically indicated, the action may be perceived as against the child's will. However, moral injury is far more likely from severe, egregious violations of patient autonomy, such as conducting invasive medical procedures without consent. Logically, we know children will cry and object to many medical treatments, but obtaining consent from both the child and the parent is recommended whenever possible. Consent to treatment requires permission from the child's legal representative and, if possible, assent from the child ( Tait & Hutchinson, 2018). The accumulation of such experiences that challenge clinicians’ duty to respect patient autonomy may eventually lead to moral injury.

## Nonmaleficence

The principle of nonmaleficence is captured by the Latin maxim, *primum non nocere:* “above all, do no harm.” It has been estimated that medical error is the third leading cause of death in the United States ( Makary & Daniel, 2016). While the potential to reduce these errors is debated, common preventable harms include medication adverse events, central line infections, and thromboembolisms ( Nabhan *et al*., 2012). With increasing ability to treat patients comes increasing opportunity to harm patients as systems become more complex. Most clinicians are very aware and regularly reminded of these statistics; however, the seemingly futile efforts to try and reduce the incidence of these harms are troublesome. Moral injury may result when there are significant lapses in nonmaleficence, such as knowingly and routinely failing to follow safety protocols. Bureaucratic and administrative interference, well intended or not, can hamper efforts by physicians and nurses to decrease harm, leading to moral injury and a sense of powerlessness.

Moral distress and moral residue arise when individuals are confronted with situations where they cannot prevent harm or alleviate suffering, even though they intend to do good. These experiences can impact one's conscience and create a lasting sense of moral conflict or guilt. However, they do not necessarily imply moral injury, which occurs after a severe violation of ethical principles or a significant lapse in professional conduct. Distinguishing between moral distress and moral injury helps us understand the range of ethical challenges that individuals may face in their professional roles.

## Beneficence

With the many opportunities to harm a patient in mind, we must also remember that patients come to clinicians for improvement or restoration of their health, which leads to the principle of beneficence. The commitment to helping others is the driving force amongst healthcare workers, and to accomplish this goal, there must be a net benefit over harm ( Gillon, 1994). Decisions on diagnostic pathways, treatment plans, and societal policies all must balance the benefit versus harms, and these balances also must be made in the context of the patient’s values.

Beneficence, when compromised, creates numerous conflicts in medicine that can result in moral injury. When the cost of proper medical care exceeds the ability of an individual patient to pay, beneficence can be compromised. Substantial moral injury may occur due to significant, unjustified lapses in beneficence, such as denying a life-saving treatment due to inability to pay.

Pharmaceutical pricing is a common cause of this moral compromise. For example, many patients with atrial fibrillation will benefit from changing their warfarin prescription to a newer, direct oral anticoagulant such as apixaban. However, the up-front price of the newer medication prohibits them from changing, even though the total financial cost of the newer medication is estimated to be lower due to fewer medical complications ( Gupta *et al*., 2018). Beyond the financial impact, the negative impact on the patient’s health can be devastating. Compromising the principle of beneficence occurs when the patient cannot take the best medication because of financial limitations. Although the medical complications from the older medication will ultimately cost more money, the hard reality is that patients will take the cheaper medication because they cannot afford the up-front costs of the newer, better medication.

## Social justice

The final pillar of bioethics is social justice. Justice demands that limited resources be distributed fairly, and that patients not be discriminated against due to any number of demographic variables such as race, religion, gender identity, sexual orientation, age, or cultural background. Moral injury occurs when these ideals conflict with the hard reality of medical care where discrimination does occur, primarily along socioeconomic lines.

These complex socioeconomic disparities cause moral injury because clinicians know what their patients need and find the economic barriers to needed care to be illogical, unnecessary, and capricious. They know that not getting that nursing home bed placement will result in a bad outcome, often at a much higher cost. They know that not getting a patient with a substance use disorder necessary treatment will ultimately cost more to society, although the health care plan may save money. They have seen first-hand the elderly family member decide they would rather die than leave a large medical bill for their surviving relatives. Witnessing these events regularly doesn’t cause burnout; it causes moral injury.

Medical professionals working in medical systems and countries that rely on privately funded insurance may also experience a constant violation of the principle of social justice. For example, one study comparing a population with universal medical insurance found disparities in the care given to racial and ethnic minorities to decrease significantly or even eliminate ( Chaudhary *et al*., 2018). A similar study found that universal medical insurance ameliorated socioeconomic disparities in mortality ( Veugelers & Yip, 2003). Medical professionals working in private insurance systems who know about and trust such research studies may experience a persistent low-grade violation of their bioethics. However, moral injury will likely occur when clinicians witness persistent, deep-rooted discrimination that leads to profoundly unequal treatment. This, over time, may progress to symptomatic moral injury. The primary means of addressing such issues would be meaningful involvement in improving the larger healthcare system.

## Conclusion

Moral injury occurs when there is a significant disruption in an individual’s sense of personal morality and capacity to behave in a just manner. While minor inconsistencies and unintentional errors are common, significant violations resulting in moral distress, failure, or injury are becoming intrinsic parts of the healthcare system. The prevention of these moral vioations is accomplished by decreasing violations of the four pillars of bioethics whenever possible. Patients deserve autonomy, and we can give this to them. Although we cannot always help our patients as much as we would like, we can always help them in at least some way. We can be vigilant when taking measures to increase patient safety and decrease harm. With a deeper understanding of bioethics, clinicians may be better equipped to reflect on moral transgressions in the workplace and how these may contribute to moral health and moral compromise.

Healthcare organizations have an obligation to provide support for practitioners experiencing various levels of moral injury resulting from unavoidable adverse events. While such unavoidable harms are a reality of clinical care, practitioners should not have to bear the moral burden alone. Institutions must not only provide forums for open discussion, but also respond productively to clinician feedback.

The arguments made here are conceptual in nature. However, improving the moral health in clinicians requires objective research leading to evidence-based improvements. Studies should examine the effect of interventions focused on both individuals and the system. The effect of education, peer support, and counseling on moral health needs further investigation. Perhaps most importantly, the effect of system-wide patient care improvements on the moral well-being of clinicians should be objectively examined and quantified.

This paper aims to provide an ethical framework, but further empirical research is critical. Understanding moral transgressions is only the start of a necessary process to increase morality throughout the healthcare system. Research looking at new medical treatments alone is insufficient; the moral implications of costs, equitable distribution, and adverse side effects must also be addressed. Questions remain: Does bioethics education help clinicians identify and cope with moral transgressions? Should a discussion of morality be a standard part of reporting clinical trials? Does simply reporting conflicts of interest and institutional review board approval provide enough of a moral foundation for clinical research? Whether addressing these issues can help prevent or reduce moral injury and burnout remains unclear, highlighting an important need for further investigation.

## Data Availability

No data are associated with this article.

## References

[ref-17] Beauchamp TL Childress JF : Principles of Biomedical Ethics.(8th ed.). Oxford University Press,2019.

[ref-18] Boudreau T : The Morally Injured. *Mass Rev.* 2011;52(3/4):746–54. Reference Source

[ref-19] Campbell SM Ulrich CM Grady C : A broader understanding of moral distress. *Am J Bioeth.* 2016;16(12):2–9. 10.1080/15265161.2016.1239782 27901442

[ref-2] Chaplo SD Kerig PK Wainryb C : Development and validation of the moral injury scales for youth. *J Trauma Stress.* 2019;32(3):448–458. 10.1002/jts.22408 31162746

[ref-3] Chaudhary MA Sharma M Scully RE : Universal insurance and an equal access healthcare system eliminate disparities for Black patients after traumatic injury. *Surgery.* 2018;163(4):651–656. 10.1016/j.surg.2017.09.045 29221878

[ref-4] Entwistle VA Carter SM Cribb A : Supporting patient autonomy: the importance of clinician-patient relationships. *J Gen Intern Med.* 2010;25(7):741–745. 10.1007/s11606-010-1292-2 20213206 PMC2881979

[ref-5] Gillon R : Medical ethics: four principles plus attention to scope. *BMJ.* 1994;309(6948):184–188. 10.1136/bmj.309.6948.184 8044100 PMC2540719

[ref-6] Gupta K Trocio J Keshishian A : Real-World Comparative Effectiveness, Safety, and Health Care Costs of Oral Anticoagulants in Nonvalvular Atrial Fibrillation Patients in the U.S. Department of Defense Population. *J Manag Care Spec Pharm.* 2018;24(11):1116–1127. 10.18553/jmcp.2018.17488 30212268 PMC10398049

[ref-30] Hodgson TJ Carey LB : Moral injury and definitional clarity: betrayal, spirituality and the role of chaplains. *J Relig Health.* 2017;56(4):1212–1228. 10.1007/s10943-017-0407-z 28526912

[ref-20] Jinkerson JD : Defining and assessing moral injury: A syndrome perspective. *Traumatology.* 2016;22(2):122–130. 10.1037/trm0000069

[ref-31] Jones KA Freijah I Carey L : Moral injury, chaplaincy and mental health provider approaches to treatment: A scoping review. *J Relig Health.* 2022;61(2):1051–1094. 10.1007/s10943-022-01534-4 35290554 PMC8922078

[ref-7] Koenig HG Ames D Youssef NA : The Moral Injury Symptom Scale-Military Version. *J Relig Health.* 2018;57(1):249–265. 10.1007/s10943-017-0531-9 29196962

[ref-21] Litz BT Stein N Delaney E : Moral injury and moral repair in war veterans: a preliminary model and intervention strategy. *Clin Psychol Rev.* 2009;29(8):695–706. 10.1016/j.cpr.2009.07.003 19683376

[ref-8] Makary MA Daniel M : Medical error-the third leading cause of death in the US. *BMJ.* 2016;353: i2139. 10.1136/bmj.i2139 27143499

[ref-9] Nabhan M Elraiyah T Brown DR : What is preventable harm in healthcare? A systematic review of definitions. *BMC Health Serv Res.* 2012;12: 128. 10.1186/1472-6963-12-128 22630817 PMC3405467

[ref-10] Nickerson A Hoffman J Schick M : A Longitudinal Investigation of Moral Injury Appraisals Amongst Treatment-Seeking Refugees. *Front Psychiatry.* 2018;9:667. 10.3389/fpsyt.2018.00667 30618859 PMC6305427

[ref-11] Shay J : Moral injury. *Psychoanalytic Psychology.* 2014;31(2):182–191. 10.1037/a0036090

[ref-12] Shay J Munroe J : Group and milieu therapy for veterans with complex posttraumatic stress disorder.In: Saigh, P. A. and Bremner, J. D. eds. *Posttraumatic Stress Disorder: A Comprehensive Text.*Boston: Allyn and Bacon,1999;391–413. Reference Source

[ref-13] Stammers T : The evolution of autonomy. *New Bioeth.* 2015;21(2):155–163. 10.1179/2050287715Z.00000000070 27124963

[ref-14] Tait AR Hutchinson RJ : Informed Consent Training in Pediatrics-Are We Doing Enough? *JAMA Pediatr.* 2018;172(3):211–212. 10.1001/jamapediatrics.2017.4088 29356830

[ref-22] Tessman L : Moral distress in health care: when is it fitting? *Med Health Care Philos.* 2020;23(2):165–177. 10.1007/s11019-020-09942-7 32034572

[ref-15] Veugelers PJ Yip AM : Socioeconomic disparities in health care use: Does universal coverage reduce inequalities in health? *J Epidemiol Community Health.* 2003;57(6):424–428. 10.1136/jech.57.6.424 12775787 PMC1732477

[ref-16] Yan GW : The invisible wound: moral injury and its impact on the health of operation enduring freedom/operation iraqi freedom veterans. *Mil Med.* 2016;181(5):451–458. 10.7205/MILMED-D-15-00103 27136652

