# Supplementary material for: Moral injury and the four pillars of bioethics
Source: F1000Res. 2023 Dec 28;8:1193. Originally published 2019 Jul 26. [Version 4] doi: 10.12688/f1000research.19754.4 (PMC10904936; doi:10.12688/f1000research.19754.4)
Supplement: Peer review history

# Reviewer report: Reviewer response for version 4

**DOI:** 10.5256/f1000research.160270.r234018

**Published:** 2024-02-16

**Authors:** Jan Helge Solbakk

**Affiliations:** 1 Department of Health and Society, Centre for medical Ethics, University of Oslo, Oslo, Norway

I find the last revisions satisfactory.

Is the topic of the opinion article discussed accurately in the context of the current literature?

Partly

Are arguments sufficiently supported by evidence from the published literature?

Partly

Are all factual statements correct and adequately supported by citations?

Yes

Are the conclusions drawn balanced and justified on the basis of the presented arguments?

Partly

Reviewer Expertise:

NA

I confirm that I have read this submission and believe that I have an appropriate level of expertise to confirm that it is of an acceptable scientific standard.

# Reviewer report: Reviewer response for version 4

**DOI:** 10.5256/f1000research.160270.r234019

**Published:** 2024-01-27

**Authors:** Lindsay B Carey

**Affiliations:** 1 Department of Public Health, School of Psychology and Public Health, La Trobe University, Melbourne, Vic, Australia

The revised Version provides a clearer understanding of MI and it's relevance to bioethics - however one needs to ask the question, "So what?".  What if the decreasing of bioethical breaches does not occur?  Or to ask the question another way: What is the effect/outcome upon health care staff and patients if one or more of the bioethical principles continue to be breached? Are the cosequences truely a matter of life and/or death? Or are the bioethical priniciples and the syndrome of moral injury just being utilized for the purposes of political/industrial exploitation?  That is to say, it is important to make clear within the conclusion (or elsewhere), the practical consequences of serious adverse events and the breaching of bioethical principles - which can lead to moral distress/anxiety and, if not resolved, ultimately can lead to a moral injury that can result in suicidal ideation / suicide - which has already been noted within the research literature i.e., consider the reviews [ref-1,2]

Is the topic of the opinion article discussed accurately in the context of the current literature?

Partly

Are arguments sufficiently supported by evidence from the published literature?

Partly

Are all factual statements correct and adequately supported by citations?

Partly

Are the conclusions drawn balanced and justified on the basis of the presented arguments?

Partly

Reviewer Expertise:

NA

I confirm that I have read this submission and believe that I have an appropriate level of expertise to confirm that it is of an acceptable scientific standard.

## References

[1] : A Review of Research on Moral Injury and Suicide Risk. *Current Treatment Options in Psychiatry* .2023;10(3) : 10.1007/s40501-023-00293-7 259-287 10.1007/s40501-023-00293-7

[2] : Examining the Association Between Moral Injury and Suicidal Behavior in Military Populations: A Systematic Review. *J Relig Health* .2023;62(6) : 10.1007/s10943-023-01885-6 3904-3925 10.1007/s10943-023-01885-6 37592186

# Reviewer report: Reviewer response for version 3

**DOI:** 10.5256/f1000research.156850.r213206

**Published:** 2023-10-19

**Authors:** Jan Helge Solbakk

**Affiliations:** 1 Department of Health and Society, Centre for medical Ethics, University of Oslo, Oslo, Norway

I have had a thorough read of version 3 of this paper, and I have still doubts about this paper, and notably, for two additional reasons which I did not address while reading a previous version:

1. The authors seem to medicalize moral injury, by putting a diagnostic label on it. I advise the authors to make it explicit that moral injury cannot be reduced to a medical diagnosis because the phenomenon of moral injury extends beyond what can be captured in medical and psychological terms and for which a cure exists.
2. The examples of situations in the health care context that might cause moral injury the authors make use of are not convincing examples. More work needs to be done to identify suitable health care situations.

Is the topic of the opinion article discussed accurately in the context of the current literature?

Partly

Are arguments sufficiently supported by evidence from the published literature?

Partly

Are all factual statements correct and adequately supported by citations?

Yes

Are the conclusions drawn balanced and justified on the basis of the presented arguments?

Partly

Reviewer Expertise:

Moral philosophy and bioethics

I confirm that I have read this submission and believe that I have an appropriate level of expertise to confirm that it is of an acceptable scientific standard, however I have significant reservations, as outlined above.

# Response

**Published:** 2023-10-20

**Authors:** Thomas F Heston

**Affiliations:** Washington State University, USA

Thank you for your comments. I believe I have addressed all recommendations and appreciate your time and effort reviewing our article.

# Reviewer report: Reviewer response for version 2

**DOI:** 10.5256/f1000research.155752.r208621

**Published:** 2023-09-29

**Authors:** Lindsay B Carey

**Affiliations:** 1 Department of Public Health, School of Psychology and Public Health, La Trobe University, Melbourne, Vic, Australia

The authors seemed to have decided not to include a number of my comments, particularly about acknowledging MI being a bio-psycho-social-spiritual syndrome; fair enough, I can understand this as the health sector is only catching up to where the veteran sector has been for 20 years. However, I strongly advise that the authors include a reference to the recent review undertaken by Phoenix Trauma Centre (Jones *et al.*, 2022 ^1^) regarding various treatments for MI as the paragraph below sits awkwardly without supporting evidence/citation.

 "The optimal treatment of moral injury remains unclear, just like the diagnosis of moral injury. Proposals to treat moral injury in medical professionals include participating in support groups, building personal character, and personal reflection by keeping a diary. The inclusion of standard treatments for post-traumatic stress disorder in veterans suffering from moral injury has also been proposed" (Jones *et al.*, 2022).

 I do like the association with the bioethical principles - which has been done with multiple topics before, but valuable to have with regard to MI.

Is the topic of the opinion article discussed accurately in the context of the current literature?

Partly

Are arguments sufficiently supported by evidence from the published literature?

Partly

Are all factual statements correct and adequately supported by citations?

Partly

Are the conclusions drawn balanced and justified on the basis of the presented arguments?

Partly

Reviewer Expertise:

Bioethics, Moral Injury

I confirm that I have read this submission and believe that I have an appropriate level of expertise to confirm that it is of an acceptable scientific standard, however I have significant reservations, as outlined above.

## References

[1] : Moral Injury, Chaplaincy and Mental Health Provider Approaches to Treatment: A Scoping Review. *J Relig Health* .2022;61(2) : 10.1007/s10943-022-01534-4 1051-1094 10.1007/s10943-022-01534-4 35290554 PMC8922078

# Response

**Published:** 2023-10-20

**Authors:** Thomas F Heston

**Affiliations:** Washington State University, USA

Thank you for your helpful comments. I believe that we have responded thoughtfully to your comments. We greatly appreciate your time and effort providing peer review.

# Reviewer report: Reviewer response for version 1

**DOI:** 10.5256/f1000research.21667.r65320

**Published:** 2020-07-13

**Authors:** Jan Helge Solbakk

**Affiliations:** 1 Department of Health and Society, Centre for medical Ethics, University of Oslo, Oslo, Norway

This is a very short and well written paper. But the paper would have benefited from further substantiation by relating the concept of moral injury to the concepts of moral failure, moral residue and moral distress.

 Here are some references the authors are advised to consult:

- Lisa Tessman, Moral distress in health care: when is it fitting? Medicine, Health Care and Philosophy (2020) 23:165–177 https://doi.org/10.1007/s11019-020-09942-7. ^1^
- Boudreau, Tyler. 2011. The morally injured. The Massachusetts Review 52(3/4): 746–754. ^2^
- Campbell, Stephen, Connie Ulrich, and Christine Grady. 2016. A broader understanding of moral distress. The American Journal of Bioethics 16(12): 2–9. ^3^
- Tessman, Lisa. 2015. Moral failure: On the impossible demands of morality. New York: Oxford University Press. ^4^
- Williams, Bernard. 1973. Ethical consistency. In Problems of the self, ed. B. Williams, 166–186. Cambridge: Cambridge University Press. ^5^

In addition, I advice the authors to consult the literature on adverse events in health care that are impossible to predict or prevent and which may cause moral distress, burnout and moral injury. That is, the fact that less than 50% of all adverse events in health care are possible to predict and prevent (of which a significant minority causes permanent disability, 7%, or death, 7%), is a painful reminder of the prevalence of unavoidable normative ignorance in health care and the importance of learning to live through moral failure caused by such events. For this, see e.g:

- Rafter, N., Hickey, A., Condell, S. et al. (2015). Adverse events in health care: learning from mistakes. QJM: An International Journal of Medicine, 108, 4: 273–277, and De Vries, E.N., Ramrattan, M.A., Smorenburg, S.M. et al. (2008). The incidence and nature of in- hospital adverse events: a systematic review. Qual Saf Health Care,17: 216-223. ^6^

Finally, the authors are advised to focus more on the problem of moral failure and injury among health care workers. In the present version of the paper the main focus is on the patient's experience of moral injury.

Is the topic of the opinion article discussed accurately in the context of the current literature?

Partly

Are arguments sufficiently supported by evidence from the published literature?

Partly

Are all factual statements correct and adequately supported by citations?

Yes

Are the conclusions drawn balanced and justified on the basis of the presented arguments?

Partly

Reviewer Expertise:

Bioethics

I confirm that I have read this submission and believe that I have an appropriate level of expertise to confirm that it is of an acceptable scientific standard, however I have significant reservations, as outlined above.

## References

[1] : Moral distress in health care: when is it fitting?. *Med Health Care Philos* .2020;23(2) : 10.1007/s11019-020-09942-7 165-177 10.1007/s11019-020-09942-7 32034572

[2] : The morally injured. *The Massachusetts Review* .2011;

[3] : A Broader Understanding of Moral Distress. *Am J Bioeth* .16(12) : 10.1080/15265161.2016.1239782 2-9 10.1080/15265161.2016.1239782 27901442

[4] : Moral failure: On the impossible demands of morality. *New York: Oxford University Press* .2015;

[5] : Ethical consistency In Problems of the self, ed. B. Williams. *Cambridge: Cambridge UniversityPress.* .1973;

[6] : Adverse events in healthcare: learning from mistakes. *QJM* .2015;108(4) : 10.1093/qjmed/hcu145 273-7 10.1093/qjmed/hcu145 25078411

# Response

**Published:** 2023-09-06

**Authors:** Thomas F Heston

**Affiliations:** Washington State University, USA

Thank you again for your time and effort in helping improve this article. I apologize for the delayed response, which was unavoidable due to a severe, prolonged illness. I believe this article remains relevant. I have attempted to fully address all of the issues raised about the different aspects of moral compromise, not just moral injury. You have made me think more deeply about this issue and I appreciate that. I am hopeful this revised version meets your approval so that it can be indexed, as this remains an important topic. As a clinician, I see a lot of focus on clinical trials and "evidence-based medicine" but the effect of these technological advances on our shared morality is only rarely discussed. This is an important topic. Thanks again.

# Reviewer report: Reviewer response for version 1

**DOI:** 10.5256/f1000research.21667.r51660

**Published:** 2019-07-26

**Authors:** Lindsay B Carey

**Affiliations:** 1 Department of Public Health, School of Psychology and Public Health, La Trobe University, Melbourne, Vic, Australia

This is an innovative and valuable consideration/discussion of moral injury (MI) in light of the key bioethical principles - both of which are used to justify the political issue of employee burnout within the clinical context.

 Given the current literature however, Shay's definition of MI (considered valuable but now too simplistic) which is used as the basis for this article, is no longer the dominant definition of moral injury since (for example) the work of Litz *et al.* (2009) ^1^, or Jinkerson (2016) ^2^, or Carey & Hodgson (2017). ^3^ It is important to note, that since Shay's definition, there have been at least 17 different definitions of Moral Injury (refer Hodgson & Carey, 2017 ^3^) and currently the most comprehensive synthesized version is that of Carey & Hodgson, 2018; *Frontiers in Psychiatry* ^4^ which needs to be noted by the authors of this article, indicating that there are other MI definitions but few utilize a holistic bio-psycho-social-spiritual paradigm to define or consider MI. 

 Most of the statements within the article are sufficiently supported; however, I think it important to cite Beauchamp and Childress (2013) ^5^ with regard to biomedical ethics and the bioethical principles (not just Beauchamp).

 Further, it can be argued that the real issue of MI within the medical/clinical context (in light of the more complex definitions of MI) should actually be due to a clinician suffering "a trauma related syndrome caused by the physical, psychological, social and spiritual impact of grievous moral transgressions, or violations, of an individual's deeply-held moral beliefs and/or ethical standards due to: (i) an individual perpetrating, failing to prevent, bearing witness to, or learning about inhumane acts which result in the pain, suffering or death of others, and which fundamentally challenges the moral integrity of an individual, organization or community, and/or (ii) the subsequent experience and feelings of utter betrayal of what is right caused by trusted individuals who hold legitimate authority" (Carey & Hodgson, 2018).

 It other words it can be argued that as a result of breaches of fundamental bioethical principles that "...grievous moral transgressions, or violations, of an individual's deeply-held moral beliefs and/or ethical standards" will occur, resulting in a moral injury (Carey & Hodgson, 2018, p. 2). Then it should be explained that "A moral injury can eventuate as a result of one or two types of occurrences, namely when (i) an individual perpetrates, fails to prevent, bears witness to, or learns about inhumane acts which result in the pain, suffering or death of others, and which fundamentally challenges the moral integrity of an individual, organization or community, and/or (ii) the subsequent moral injury experience and feelings of utter betrayal of what is right, caused by trusted individuals who hold legitimate authority" (Carey & Hodgson, 2018, p.2). 

 To shift too far from such a definition/explanation would mean that it is not really a complex 'moral injury' at all - but rather a 'superficial' incident that conflicts with professional bioethics. Put simply, the more advanced / complex definitions of moral injury should be utilised and will actually co-align a lot easier with the bioethical principles.

 The conclusions are somewhat justified on the basis of the presented arguments; however, it is somewhat of an assumption to conclude that ....a firm understanding of bioethics ....will prevent recurrent MI! This is doubtful - indeed t'would be like saying that a better understanding of bioethics will prevent the effects of witnessing a trauma related incident (e.g., a murder). Highly improbable!

 There is also no evidence provided to indicate/justify that a better recognition of the connection between bioethics and MI will decrease burnout! Indeed one can speculate that better recognition might actually increase one's stress, and increase the chances of subsequent burnout! (Not decrease burnout!). The most one could argue (in the absence of solid evidence) would be that "a better understanding of the effects of breaching bioethical principles within the work place, and the possible correlation with experiencing a moral injury, may explain feelings of recurrent burnout"... but it certainly would NOT prevent MI nor unlikely to prevent injuries. The conclusion needs to be edited as well as adding a note for empirical research to be undertaken with regard to MI and clinician burnout in the clinical context.

Is the topic of the opinion article discussed accurately in the context of the current literature?

Partly

Are arguments sufficiently supported by evidence from the published literature?

Partly

Are all factual statements correct and adequately supported by citations?

Partly

Are the conclusions drawn balanced and justified on the basis of the presented arguments?

Partly

Reviewer Expertise:

Bioethics, Moral Injury

I confirm that I have read this submission and believe that I have an appropriate level of expertise to confirm that it is of an acceptable scientific standard, however I have significant reservations, as outlined above.

## References

[1] : Moral injury and moral repair in war veterans: a preliminary model and intervention strategy. *Clin Psychol Rev* .2009;29(8) : 10.1016/j.cpr.2009.07.003 695-706 10.1016/j.cpr.2009.07.003 19683376

[2] : Defining and assessing moral injury: A syndrome perspective. *Traumatology* .2016;22(2) : 10.1037/trm0000069 122-130 10.1037/trm0000069

[3] : Moral Injury and Definitional Clarity: Betrayal, Spirituality and the Role of Chaplains. *J Relig Health* .2017;56(4) : 10.1007/s10943-017-0407-z 1212-1228 10.1007/s10943-017-0407-z 28526912

[4] : Chaplaincy, Spiritual Care and Moral Injury: Considerations Regarding Screening and Treatment. *Front Psychiatry* .2018;9: 10.3389/fpsyt.2018.00619 619 10.3389/fpsyt.2018.00619 30568605 PMC6290645

[5] : Principles of Biomedical Ethics. *Oxford University Press* .2013;

# Response

**Published:** 2019-07-26

**Authors:** Thomas F Heston

**Affiliations:** Washington State University, USA

I appreciate the comments from the reviewer and in general agree. In other groups outside of health care providers, moral injury is becoming more precisely defined. However, the definition and implications of moral injury in health care professionals currently remains vague. With this perspective paper, we aim to stimulate investigation into the relationship between a violation of well established bioethical principles and moral injury. We remain convinced that moral injury, both minor and large, regularly affects medical professionals, and that there most likely is a strong relationship to the four pillars of bioethics. Nevertheless, more research and investigation clearly is indicated. Again, the comments from the reviewer are thorough and greatly appreciated.

# Response

**Published:** 2019-07-27

**Authors:** Lindsay B Carey

**Affiliations:** Palliative Care Unit, La Trobe University, Australia

Dear Article Authors,

I concur with your "aim to stimulate investigation into the relationship between a violation of well established bioethical principles and moral injury" and "that moral injury.... regularly affects medical professionals, and that there most likely is a strong relationship between (breaches of) the four pillars of bioethics" and moral injury - Indeed this seems logical and most viable. However my concern is that, currently your understanding of MI "remains vague" and this is understandable because some researchers and even yourselves, have based their understanding of MI on a basic definition.  Except for those who wish MI to remain vague/basic for their own purposes, the research regarding MI, demonstrates that MI is far more complex than originally conceived.  

I think it is important to note that on the one hand you opt for a simple definition of MI, yet one of your own article statements aligns with more complex definitions: *"When a physician, nurse, or other health care provider participates in, or witnesses a violation of, one or more of these core principles, moral injury occurs"*.  I am simply suggesting: (1) the correlation between violations of bioethical principles and a MI or a potential moral injury event (PMIE), seems logical and would unquestionably affect clinician morale, however any correlation between bioethical principles and MI requires a more complex definition of MI. (2) There is no need for another definition of MI specific to clinicians  - this would simply muddy the waters - there are already several comprehensive definitions (Litz et al, Jinkerson and a combination of Shays and others by Carey & Hodgson) as already noted in my earlier review - which are all based on empirical research/case studies.  If there is no correlation with these more complex definitions, then perhaps it is not moral injury to which you are referring, but something entirely different.

To be sure however, I support your argument/logic about bioethical principles regularly being breached in the health care context which could result in a moral injury for clinicians, however MI is complex and therefore requires a more comprehensive definition - which in my view would actually support your investigation into the relationship between a violation of well established bioethical principles and moral injury.

# Response

**Published:** 2023-09-06

**Authors:** Thomas F Heston

**Affiliations:** Washington State University, USA

Thank you again for your time and effort in helping improve this article. I apologize for the delayed response, which was unavoidable due to severe, prolonged illness. I believe this article remains relevant. I have attempted to fully address all of the issues raised. Thank you- the article is significantly improved. I am hopeful this meets your approval so that it can be indexed, as this remains an important topic.
